# Rising Influence of Climate on the Distribution of Black‐Necked Cranes (*Grus nigricollis*) on Tibetan Plateau

**DOI:** 10.1002/ece3.72756

**Published:** 2025-12-28

**Authors:** Le Yang, Ting Wu, Waner Liang, Qin Zhu, Lei Xu, Jia Guo, Cai Lu, Qing Zeng, Mary Anne Bishop, Li Wen, Yifei Jia, Guangchun Lei

**Affiliations:** ^1^ School of Ecology and Nature Conservation Beijing Forestry University Beijing China; ^2^ Key Lab of Biological Resources and Biosecurity of Xizang Autonomous Region, Institute of Plateau Biology of Xizang Autonomous Region Lhasa China; ^3^ Center for East Asian‐Australasian Flyway Studies Beijing Forestry University Beijing China; ^4^ Nanjing University Nanjing China; ^5^ International Crane Foundation Baraboo Wisconsin USA; ^6^ Prince William Sound Science Center Cordova Alaska USA; ^7^ Science and Insights Division, Department of Climate Change, Energy, the Environment and Water Parramatta New South Wales Australia

**Keywords:** black‐necked crane, climate change, species distribution model, wintering habitat

## Abstract

The Qinghai‐Tibet Plateau (QTP) wetland ecosystem is undergoing significant changes because of global climate change, which is likely to affect the distribution of waterbirds. To enhance our understanding of these ecosystems and their waterbirds, we used the black‐necked crane ( *Grus nigricollis* ) as an indicator and examined shifts in the distribution of its suitable habitats. We analyzed field‐collected location data and citizen‐science data from mid‐southern Tibet for 2006 and 2020 and employed the Species Distribution Model (SDM) to assess changes in suitable wintering habitats. Our findings revealed that between 2006 and 2020, suitable habitats for wintering black‐necked cranes expanded overall, though some suitable areas were lost. The expansions are mainly distributed around Qinghai Lake and the northern part of the Yunnan‐Guizhou Plateau. Notably, the variable importance scores from 70.7% land use and land cover in 2006 to 56.9% in 2020, with climate variables such as G‐bio8 and G‐bio18 gaining prominence. As the black‐necked crane's range expands, there may be an increase in overlap in its wintering areas. We recommend not only strengthening the management of existing protected areas but also enhancing research on habitat connectivity from a Central Asian Flyway perspective to better address the impacts of climate change.

## Graphical abstract

The Qinghai‐Tibet Plateau (QTP) wetland ecosystem is undergoing significant changes because of global climate change, which is likely to affect the distribution of waterbirds. To enhance our understanding of these ecosystems and their waterbirds, we used the black‐necked crane ( *Grus nigricollis* ) as an indicator and examined shifts in the distribution of its suitable habitats. We recommend not only strengthening the management of existing protected areas but also enhancing research on habitat connectivity from a Central Asian Flyway perspective to better address the impacts of climate change.

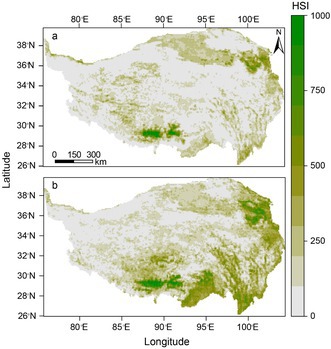

## 1 Introduction

The IPCC Sixth Assessment Report (AR6) states that the global surface temperature has risen 1.99 (likely range: 0.95°C–1.20°C) during 2011–2020 relative to 1850–1900 (IPCC 2021), with temperatures continuing to rise (Cai et al. 2022). This warming effect is particularly pronounced in plateau regions, where higher altitudes amplify climate change effects, a phenomenon known as elevation‐dependent warming (Pepin et al. 2015; Palazzi et al. 2017). On the Qinghai–Tibet Plateau (QTP), the active layer thickened by 0.15–0.50 m and ground temperature at 6 m depth rose by 0.10°C–0.30°C between 1961 and 2001 (Cheng and Wu 2007). As a result, the QTP has emerged as one of the most affected regions globally by climate change (Ran et al. 2018). As the highest and largest plateau in the world, the Qinghai‐Tibet Plateau, with its numerous endemic species, is one of the important alpine biodiversity hotspots (Liu et al. 2014; Zhang, Jiang, et al. 2025). Continued warming is expected to have profound impacts on wetland ecosystems, altering hydrological processes, vegetation succession, and wetland‐dependent species' habitats (Huang et al. 2016; Xu et al. 2019; Jia et al. 2021). These effects are compounded by rising human activity on the plateau, which intensifies habitat degradation, leading to shifts or loss of suitable habitats for waterbirds and other species (Newbold et al. 2016).

The black‐necked crane ( *Grus nigricollis* ) breeds chiefly on the QTP and winters in lower‐altitude regions, including Yunnan (China), Bhutan, and northern India (IUCN 2025). Its population has shown a steady increase in recent years because of effective conservation efforts, including CEPA (Communication, Education, Participation, and Awareness) initiatives. In 2020, the International Union for Conservation of Nature (IUCN) listed the black‐necked crane as Near Threatened (NT) on the basis of global population assessments (Liu and Chen 2000; IUCN 2025). However, the species continues to face significant survival threats—such as climate warming‐induced wetland degradation (e.g., loss of shallow wetlands due to permafrost thaw and altered hydrology) and ongoing human pressures including infrastructure development and land‐use change (Yang et al. 2023; Jiang et al. 2025; Wang and Shen 2020). As a flagship species of the plateau, changes in their distribution and population trends serve as indicators of habitat shifts and ecosystem health (Hou et al. 2021; Niemi et al. 1997). Nevertheless, in the context of global climate warming, the dynamics of suitable habitats for the black‐necked crane on the Qinghai–Tibet Plateau have not yet been clearly elucidated.

The Yarlung Zangbo River Basin in mid‐southern Xizang (Tibet) is the largest wintering ground for black‐necked cranes (Song et al. 2014). Previous studies (e.g., Song et al. 2014; Han et al. 2018) have highlighted the ecological importance of this region for black‐necked cranes and the challenges posed by land‐use change. Between 1980 and 2020, the wintering population in this region increased from approximately 900 individuals to over 5147 (Yang et al. 2023). Meanwhile, the Yarlung Zangbo River Basin, which supports over one‐third of Tibet's population (approximately 1.13 million people in 2018) despite covering only about 5.5% of the region's area, has experienced rapid economic growth (GDP reaching CNY 69.48 billion in 2018), escalating population pressure, expansive urban and agricultural development, and significant land‐use changes—converted woodland, grassland, and wetlands into residential and agricultural land—resulting in ecological degradation and fragmentation of habitats (Hao et al. 2023). Balancing conservation and development in this area is critical for sustaining the species in the future. Although prior studies have predicted the habitat distribution of the black‐necked crane (Li et al. 2022; Han et al. 2018), quantitative evaluations of the combined impacts of climate warming and human activities on its suitable habitats—particularly across long temporal scales—remain scarce.

Additionally, elevation‐dependent warming disproportionately affects alpine ecosystems, altering wetland hydrology and vegetation composition (Palazzi et al. 2017; Mao et al. 2018). For example, warmer temperatures can lead to permafrost thaw, shifts in snowmelt timing, and changes in wetland water availability (Liu and Chen 2000; Yang et al. 2023; Jin et al. 2021), which directly impact waterbird habitats. Although climatic variables, such as temperature and precipitation, also indirectly influence waterbird habitat distribution by altering wetland structure and food availability (Li et al. 2022; Han et al. 2018). Yet, it is still uncertain whether the importance of climatic factors and land‐use types for black‐necked crane habitats has remained constant or undergone shifts in response to intensified climate warming. Clarifying whether the key determinants of suitable habitats for the black‐necked crane have changed is essential for informing conservation strategies. Species distribution models (SDMs) are used to estimate the potential spatial distribution of species by establishing statistical relationships between observed occurrence data and relevant environmental variables (Guisan et al. 2013; Murphy and Smith 2021; Elith and Leathwick 2009). In this study, we employed the Random Forest (RF) model, a robust ensemble learning algorithm known for its high predictive accuracy and ability to handle complex, nonlinear relationships (Norberg et al. 2019). RF is particularly suitable for ecological modeling because of its resistance to overfitting and its effectiveness with mixed‐type predictors. Key environmental variables used in our model include climatic conditions (e.g., temperature and precipitation), soil features, and land use (e.g., Land use and Land cover).

Building on these insights, we formulated the following two scientific questions to guide our study: how has the suitable habitat of the black‐necked crane on the Qinghai–Tibet Plateau gained or lost between the study periods? what are the main factors driving the changes in the distribution of suitable habitats? and how have their relative contributions shifted over time?

To address these issues, in this paper, we modeled the distribution of black‐necked cranes in their primary wintering habitats on the QTP over two time periods (i.e., 2006 and 2020). The specific objectives were to: (1) quantify the gain and loss of the suitable habitat of black‐necked cranes on the QTP; and (2) identify and quantify the main factors driving changes in habitat distribution. Understanding the impacts of climate change on the black‐necked crane's wintering habitats is crucial for guiding conservation strategies in this sensitive region. As a flagship species of the QTP, the conservation of black‐necked cranes reflects broader ecological challenges and priorities in this high‐altitude ecosystem. The results of this study will provide insights into the potential risks to waterbird populations under different climate scenarios and inform adaptive management plans to safeguard critical wetland habitats. Additionally, the findings can contribute to balancing the competing needs of biodiversity conservation and socio‐economic development in rapidly growing regions like the Yarlung Zangbo River Basin. By identifying priority areas for habitat protection and restoration, this research will support the development of effective, evidence‐based conservation policies for black‐necked cranes and the broader plateau ecosystem.

## 2 Methods

### 2.1 Study Area

**FIGURE 1:**
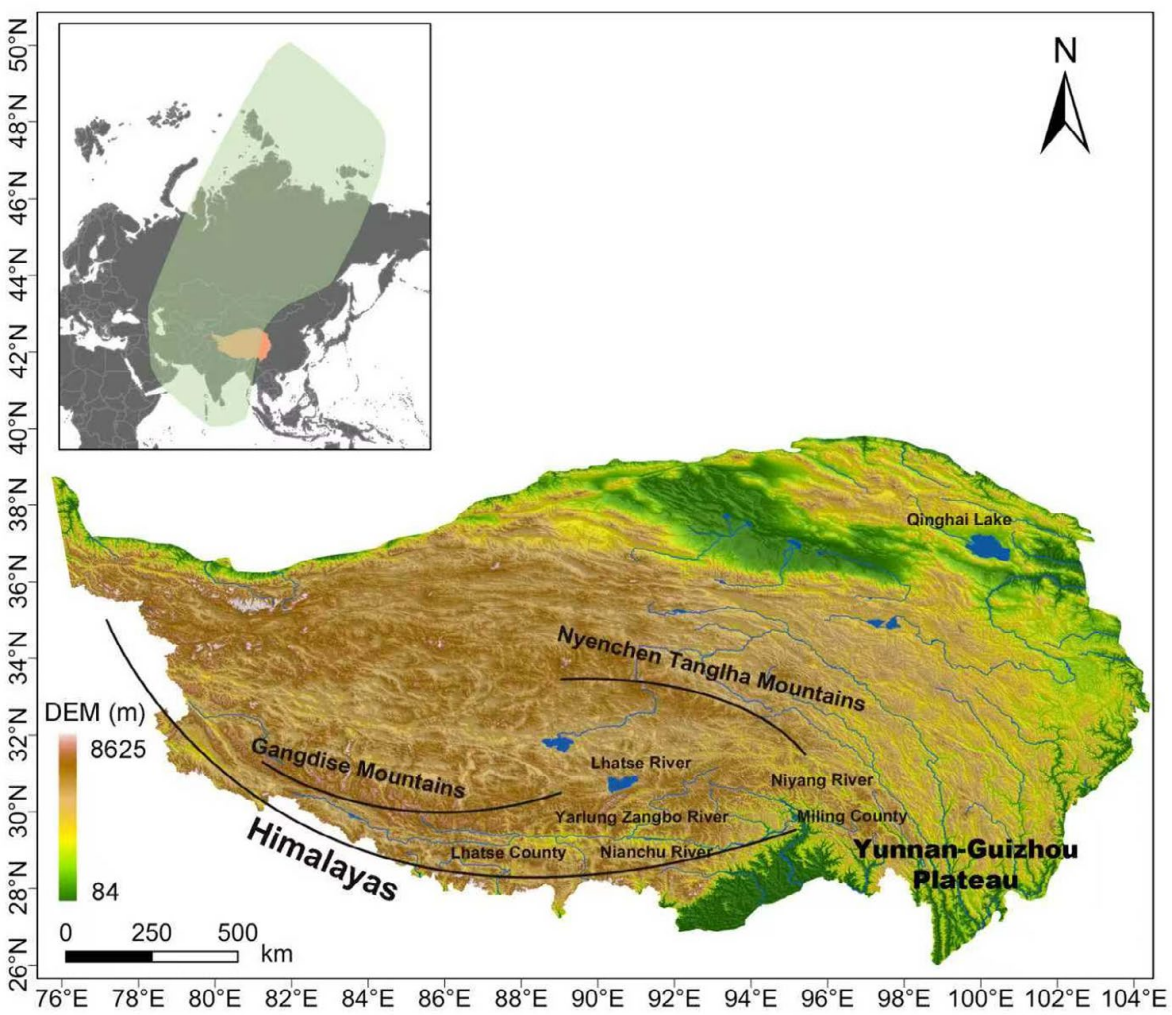
Study area: the green area in the inset map marks the Central Asian Flyway. The orange in the insert map marks the study area.

The study area (26°‐40° N, 73°‐104° E, Figure 1) covers the entire QTP, with an average elevation of over 4000 m. Within the study area, the middle reach of the Yarlung Zangbo River and its three tributaries (Lhasa River, Nianchu River, and Niyang River) represent one of the core breeding and foraging areas of the species (Tianchou 1999; Cui and Graf 2009). The study area extended from Lazi County in Shigatse City in the west to Milin County in Nyingchi City in the east, with a length of 685 km; it reached the plateau lake basin at the northern Himalayas in the south and was bounded by the Gangdise Mountains‐Nyenchen Tanglha Mountains in the north, with a width of about 220 km (Figure 1).

There were great spatial variations in climate in the study area, which directly shape the wetland and farmland habitats critical for the black‐necked crane. Together with the tributary Lhasa River and the Nianchu River Basin, the middle Yarlung Zangbo River presents a plateau temperate monsoon arid or semi‐arid climate, with cold winters and mild summers. Distinct wet and dry seasons govern the formation of wetlands and seasonal water bodies, providing critical breeding and foraging habitats. The region also receives abundant solar radiation but has a short frost‐free period, which restricts vegetation growth and influences crane breeding patterns (Zhao et al. 2021). In contrast, the lower reaches exhibit a plateau temperate monsoon semi‐humid climate, with longer frost‐free periods, relatively higher precipitation, and higher humidity. These conditions support more stable wetlands and croplands, which serve as important wintering and stopover sites for the species. From 1957 to 2012, the air temperature of the Qinghai–Tibet Plateau increased by 2.04°C, with an average warming rate of 0.037°C per year, and the rate of warming has continued to accelerate (Mei et al. 2024). Such rapid climatic changes further intensify the vulnerability of the region's fragile ecosystems (Lu et al. 2022).

The catchment of the Yarlung Zangbo River and its three tributaries is the main traditional agricultural area in Tibet, accounting for more than 80% of the total cultivated land. Although it is a region with high mountains and canyons, there are still plains formed by river alluvium at intervals on the terrain (Cui and Graf 2009). Farms in this region have sufficient water resources with convenient irrigation and deep soil, which are suitable for the growth of a variety of crops (Zhang et al. 2022), such as Highland barley (*Hordeum vulgare var*. coeleste Linnaeus), wheat ( *Triticum aestivum* ), pea (*Pisum sativm*), and rapeseed (*Brassica capestris*), as well as the black‐necked crane with steady food sources. The agricultural production pattern is relatively stable; the grain yields have increased by 34.68% in the past 20 years, but the total amount of arable land is shrinking, and the degree of fragmentation has increased (Wang et al. 2022).

### 2.2 Bird Occurrence Data in 2006 and 2020

**FIGURE 2:**
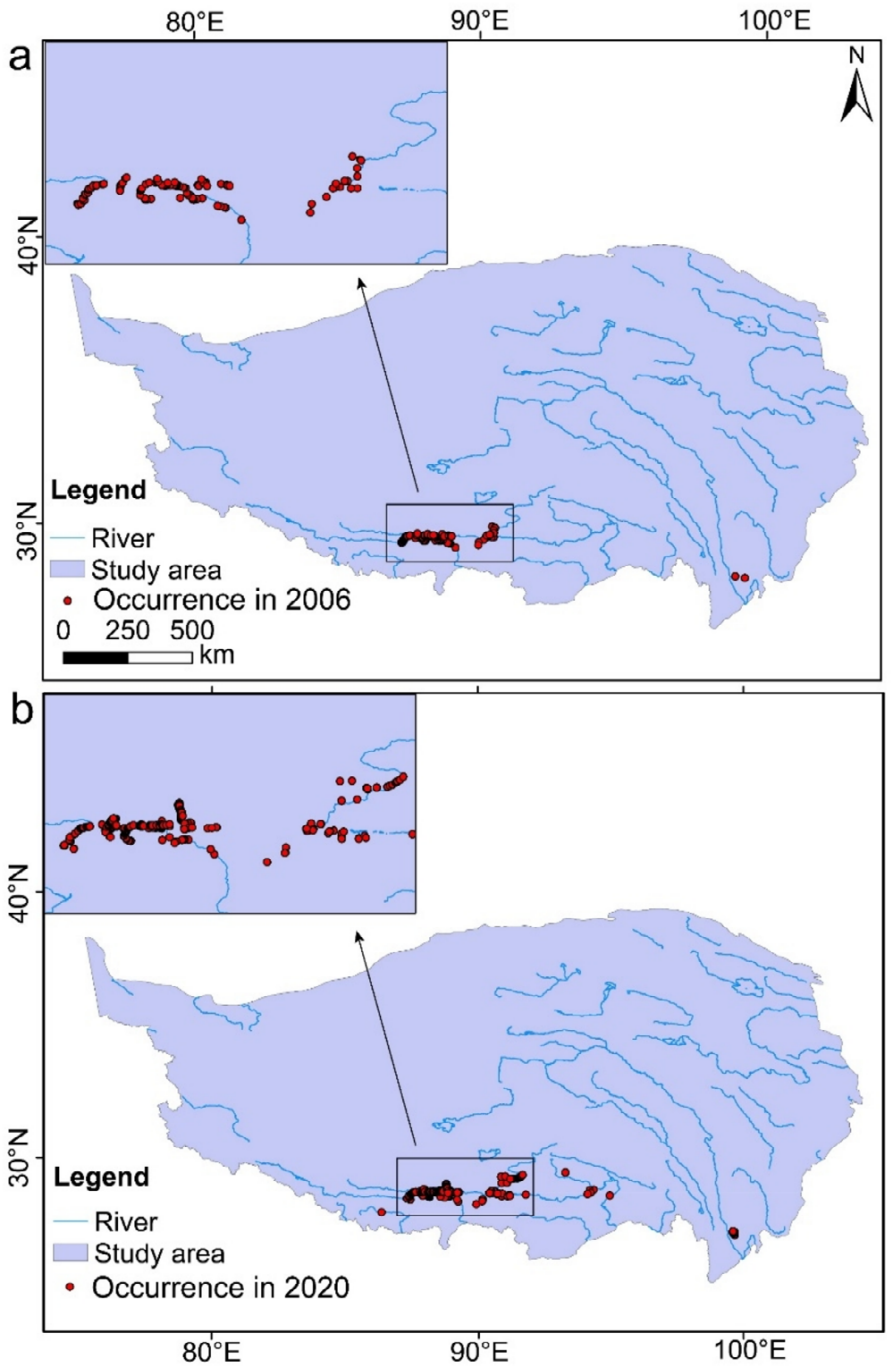
The occurrence records of wintering black‐necked crane in Tibet in 2006 (a) and 2020 (b).

We compiled two black‐necked crane occurrence datasets, one in the 2006 wintering period (November to March of the following year) and the other in the 2020 wintering period (November to March of the following year), 15 years apart. The two time points capture the rapid environmental change (including climate, soil features, and LULC) period in the Tibet Plateau. The primary sources of occurrence data were from systematic wintering bird surveys, with the complementary data colligated from citizen science databases, including China Bird Report (http://www.birdreport.cn/), eBird (https://ebird.org/home), and GBIF (https://www.gbif.org/). The surveys were jointly conducted at the middle reaches of the Yarlung Zangbo River (including the Nianchu River and Lahsa River) by Tibet Plateau Institute of Biology, the International Crane Foundation, and Beijing Forestry University. The final dataset comprises 211 valid species occurrence records from 2006 and 1056 valid species occurrence records from 2020 (Figure 2). In order to avoid spatial autocorrelation and bias in species occurrence data caused by survey methodologies, only one occurrence record was retained per grid cell (10 km^2^). Consequently, 54 species occurrence records were retained for the year 2006, and 82 records were retained for the year 2020.

### 2.3 Environmental Variables

Because of the views of changes in the species distribution and habitat that were significantly associated with climate change being obvious (Chuine 2010; Wang, Liu, et al. 2021; Perry et al. 2005), coupled with the heightened vulnerability and sensitivity of the Qinghai‐Tibet Plateau to climate change (Li et al. 2023), we selected 19 bioclimatic factors to indicate climate change, Soil Moisture Index (SMI), and land use and land cover (LULC) data to represent habitat condition. In addition to winter climate conditions (e.g., temperature and precipitation), we also included summer climatic variables in the modeling. This is because the quality of wintering habitats is not only regulated by winter climate but also by summer conditions. For instance, summer precipitation strongly influences the extent of wetlands and agricultural productivity, which in turn determine food availability and roosting environments during winter (Bai et al. 2022; Wu et al. 2020). Therefore, both winter and summer climatic factors were considered in the selection of environmental predictors.

To define bioclimatic variables, we followed the framework provided by WorldClim (https://worldclim.org/data/bioclim.html) but generated the variables using real‐time climate data specific to our study periods: 2006 (2006–2007) and 2020 (2020–2021). We named them G‐bio1 to G‐bio19. This approach ensured better alignment with the temporal scope of our analysis.

Bioclimatic variables were sourced from the ERA5‐Land Hourly dataset and processed in Google Earth Engine (GEE). Using the dismo package in R, we derived bioclimatic variables (G‐bio1–G‐bio19) from temperature and precipitation data. SMI data were sourced from the TerraClimate dataset and processed in GEE. LULC was obtained from the China Multi‐Period Land Use Remote Sensing Monitoring 30 m Dataset (all the details of environmental variables are presented in Table S1), which is constructed on the basis of artificial visual interpretation using US Landsat remote sensing images as the main information source, and represents multi‐period land use data at the national scale of China (Xinliang et al. 2018). To ensure the accuracy of the 30 m LULC data, we used the bioclimatic variables as a reference template. We then calculated the number of 30 m pixels that intersected with each 10 km^2^ grid cell and determined the proportion of 30 m pixels within each 10 km^2^ grid. For each land category, separate calculations were conducted; thus, we obtained 25 land category proportion raster data in 10 km^2^ grids. The names of each land category are presented in Figures S1 and S2.

All environmental layers were clipped to the geographic extent of the study area and resampled to a uniform resolution of 10 km^2^ using ArcMap 10.2. The 10 km^2^ resolution was chosen to balance spatial details with computational efficiency and to match the scale of environmental data. To address collinearity among predictor variables (Dormann et al. 2013), pairwise Spearman correlation coefficients were calculated. Variables with correlation coefficients exceeding |*R*| > 0.75 were excluded to ensure the selection of independent predictors, following best practices for SDM development. The environmental factors included in the calculation are presented in Table 1.

**TABLE 1:** Environmental data used for constructing SDMs.

Environmental variables	2006	2020	Source
Bioclimatic variables	G‐bio3	G‐bio3	https://cds.climate.copernicus.eu/datasets/reanalysis‐era5‐land?tab = overview
	G‐bio10	G‐bio8	
	G‐bio12	G‐bio14	
	G‐bio15	G‐bio15	
	G‐bio19	G‐bio18	
		G‐bio19	
Soil moisture index	SMI	SMI	https://www.climatologylab.org/terraclimate.html
Land use and land cover	LULC	LULC	https://www.resdc.cn/DOI/doi.aspx?DOIid=54

### 2.4 Species Distribution Model

Following the removal of duplicate occurrences and spatial filtering to retain one record per grid cell, a total of 54 and 82 species presence points were used for modeling in 2006 and 2020, respectively. Random Forest (RF) was selected to develop the SDM because of its robustness to overfitting and ability to handle complex interactions among predictors (Ramampiandra et al. 2023). Since RF models require both presence and absence data, we randomly generated corresponding pseudo‐absence points. These pseudo‐absences were generated by random sampling within the study area, ensuring spatial independence from presence points.

To evaluate the performance of our SDMs, we employed a commonly used random 80/20 split approach, where 80% of the occurrence data were used for model training and 20% for testing. This method provides a straightforward way to assess predictive accuracy and has been widely applied in ecological modeling studies. However, we acknowledge that random splitting may lead to inflated performance metrics because of spatial autocorrelation, where nearby training and testing points share similar environmental conditions. This can result in overly optimistic estimates of model performance, particularly in spatially structured datasets. To address this limitation, we have reported complementary evaluation metrics, including the Boyce Index, which is less sensitive to spatial autocorrelation and better suited for presence–pseudo‐absence data (Liu et al. 2025). In addition, we have reported the confusion matrices at the selected threshold (maximum TSS) to offer a transparent view of model classification performance. Included are prevalence, the number of presence and pseudo‐absence points, and the threshold used for binarising habitat suitability maps. Although spatially blocked cross‐validation (CV) offers a more conservative and spatially explicit validation framework, we opted to retain the random split for consistency with previous studies because of the relatively dense and geographically constrained sampling effort in our study area. We recommend that future studies consider spatially blocked CV for broader‐scale or more heterogeneous datasets.

All model training and processing were implemented in the “biomod2” package (Thuiller et al. 2025) within R 4.4.3 (R Core Team 2025), which is the most well‐known and well‐established ensemble tool in the SDM community. For species range change calculation, we first load the binary we produced during the 2006 and 2020 distribution projections. Then we calculate the change in species ranges between 2006 and 2020 using the BIO_RangeSize function (Guisan et al. 2017).

## 3 Result

### 3.1 Black‐Necked Crane Species Distribution Model in 2006 and 2020

All the repeated RF models exhibited high predictive accuracy, with a mean AUC value of 0.945, TSS of 0.809, Boyce Index of 0.547 in 2006, with a mean AUC value of 0.972, TSS of 0.881, Boyce Index of 0.500 in 2020, on the basis of the testing dataset (Table S2). Additionally, the dataset prevalence was 0.481 in 2006 and 0.491 in 2020, with presence/pseudo‐absence sample sizes of 50/54 and 79/82, respectively. The threshold used for binarizing the continuous suitability maps was 0.49. Response curves of environmental variables are presented in Figures S1 and S2.

### 3.2 Potential Suitable Habitat Distribution and Range Change for Black‐Necked Cranes Between 2006 and 2020

**FIGURE 3:**
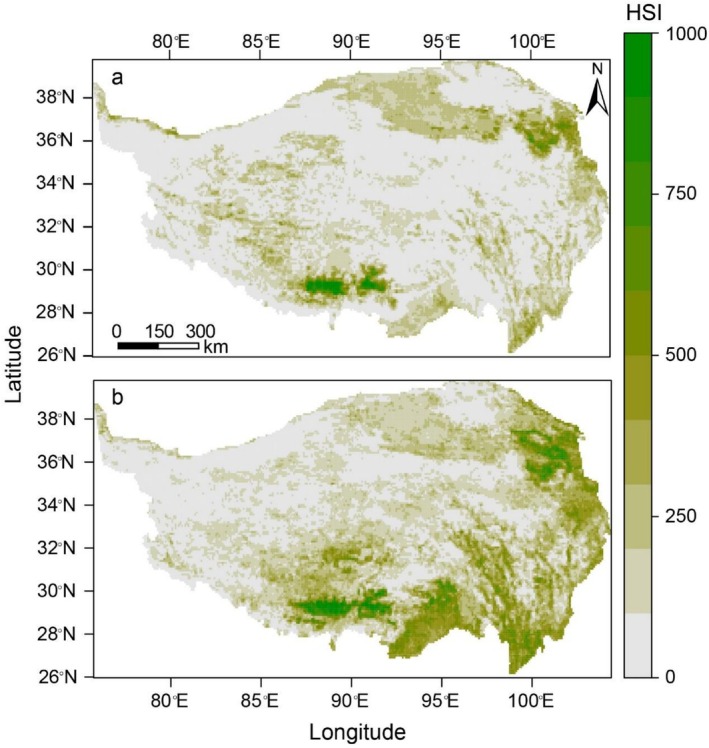
Distribution of suitable habitat for black‐necked crane in 2006 and 2020. (a) Suitable distribution area of black‐necked cranes in 2006; (b) suitable distribution area of black‐necked cranes in 2020. The gradient legend on the right indicates the habitat suitability index (HSI), and the values were converted into 0–1000 integer scale.

In 2006, the potentially suitable habitat for wintering black‐necked cranes was primarily concentrated in the Yarlung Zangbo River basin and around Qinghai Lake. By 2020, the distribution expanded to include the mainstream of the middle reaches of the Yarlung Zangbo River and extended into the Lhasa River basin. Additionally, there was a noticeable expansion of suitable habitat in the northern region of the Yunnan‐Guizhou Plateau. Between 2006 and 2020, areas of potential suitable habitat for wintering black‐necked cranes increased by 17,460 km^2^ (Table 2), reflecting an overall expansion in their wintering range (Figure 3).

**TABLE 2:** Average contribution (%) of environmental variables to the distribution probability of black‐necked crane in 2006 and 2020.

2006		2020	
Environmental variables	Contribution (%)	Environmental variables	Contribution (%)
LULC	70.7	LULC	56.9
G‐bio10	29.7	G‐bio8	23.4
SMI	11.9	G‐bio18	14.2
G‐bio15	6.2	G‐bio3	10.6
G‐bio12	5.9	SMI	9.3
G‐bio3	5.7	G‐bio19	6.3
G‐bio19	1.6	G‐bio15	5.6
		G‐bio14	3.3

**FIGURE 4:**
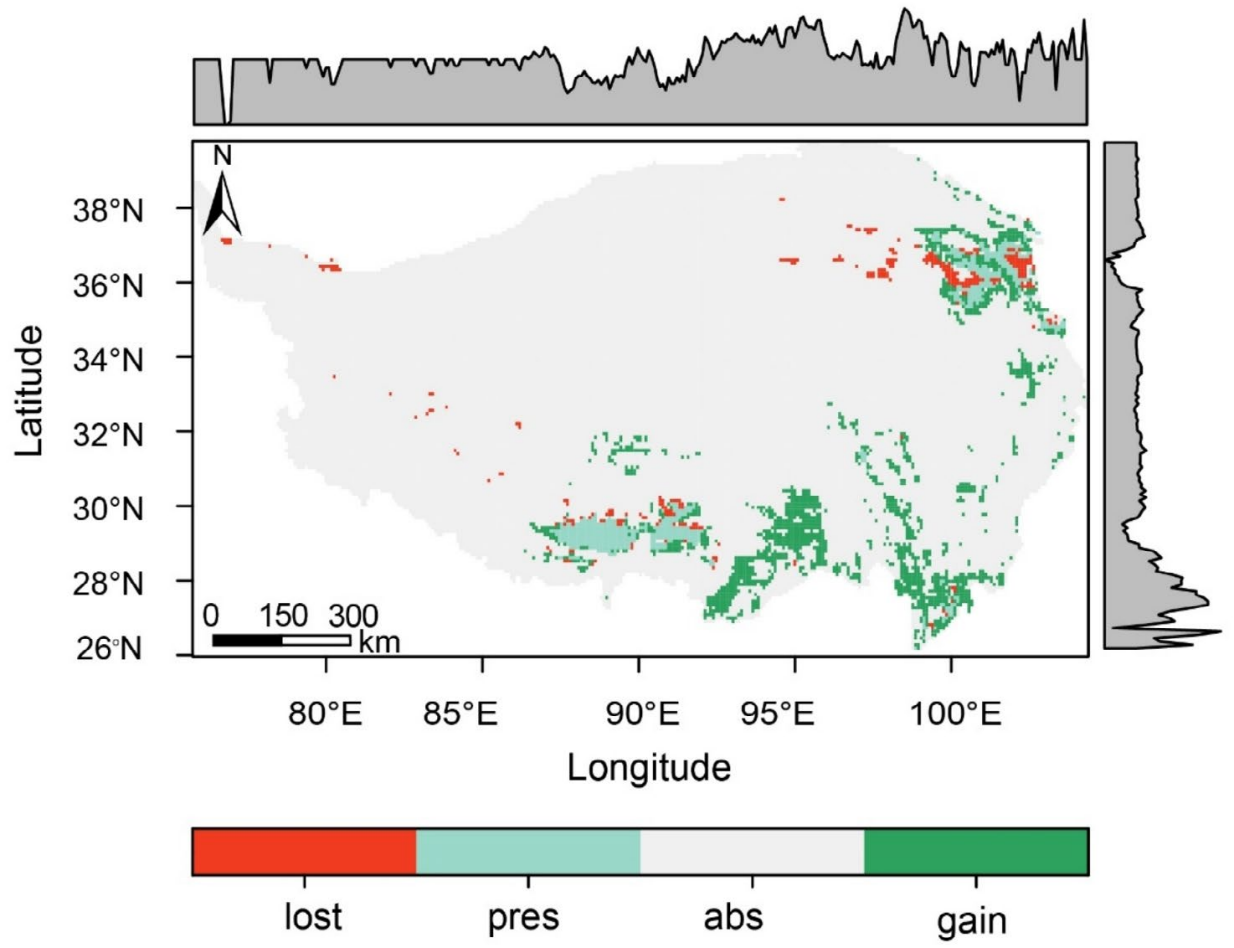
Changes in the suitable habitat for wintering black‐necked cranes between 2006 and 2020, “lost” indicates the loss of suitable habitats from 2006 to 2020, “pres” indicates stable suitable habitats over the same period, “abs” indicates areas that remained unsuitable in both years, and “gain” indicates the newly suitable habitats in 2020. The shaded areas at the top and on the right represent the distribution of the suitable black‐necked crane habitats in the direction of longitude and latitude, respectively.

Over a period of 15 years, an overall expansion of wintering black‐necked crane suitable habitats was observed (Figure 4), especially in the areas surrounding Qinghai Lake and the northern part of the Yunnan‐Guizhou Plateau. However, despite the increase in total suitable habitat, some areas of the original habitat showed signs of disappearing. In general, the area of new suitable habitat exceeded the area lost. For example, compared to the 2006 baseline, 3370 km^2^ disappeared, 17,460 km^2^ increased, and 6850 km^2^ suitable habitat remained unchanged (Table S3). From the perspective of spatial differences, the loss of suitable habitats for the black‐necked cranes mainly occurred in the northeastern part of the study area, whereas the increase mainly occurred in the southeastern part.

### 3.3 The Key Environmental Factors Affecting the Distribution of Black‐Necked Crane in 2006 and 2020

Overall, among all considered environmental factors, LULC exerted the strongest influence on the spatial distribution patterns of black‐necked cranes in both 2006 (70.7%) and 2020 (56.9%). The importance of bioclimatic variables influencing the distribution of suitable wintering habitats for black‐necked cranes varied between 2006 and 2020. In 2006, the Mean Temperature of the Warmest Quarter (G‐bio10, 29.7%) and the Soil Moisture Index (SMI; 11.9%) were identified as key environmental predictors. By contrast, in 2020, the most influential variables include the Mean Temperature of Wettest Quarter (G‐bio8, 23.4%), Precipitation of the Warmest Quarter (G‐bio18, 14.2%), and Isothermality (G‐bio3, 10.6%), indicating a shift in the relative contribution of climatic drivers over time (Table 2).

## 4 Discussion

This study investigated the changes in the suitable habitats of the black‐necked cranes over the past 15 years, as well as the dominant environmental factors influencing these changes. The findings align with and expand upon existing research, highlighting the species' sensitivity to climate dynamics and land‐use changes.

### 4.1 Climate‐Driven Habitat Shifts

The significant shifts in black‐necked crane wintering habitats between 2006 and 2020 support our scientific questions and align with other research documenting the ecological consequences of rapid warming on the Qinghai‐Tibet Plateau (Li et al. 2022). We observed poleward and altitudinal range shifts of plateau bird species, with results demonstrating eastward and upward habitat expansions.

One contributing factor may be the increase in open water area between 2013 and 2020 (Yang et al. 2023) likely driven by new thermally favorable conditions created by rapid regional warming—progressing at twice the global average rate (IPCC 2021). Warming‐induced permafrost degradation and associated hydrological changes may increase water storage in wetlands and lakes, thereby affecting habitat availability (Wu et al. 2009; Zhang, Mo, et al. 2025). Although this linkage a working hypothesis for the Yarlung Zangbo River wintering area, it is consistent with broader evidence from the QTP. As a wetland‐dependent species, the black‐neck Crane relies on areas with abundant water sources, such as shallow lakes and marshes, for habitat and foraging. The expansion of lakes is expected to increase their suitable habitats (Yang et al. 2023). In our study, the most significant habitat expansions occurred around Qinghai Lake and the Yarlung Zangbo River Basin. Previous studies have confirmed expansion in these areas (Meng et al. 2024; Xiao et al. 2025), with abrupt shifts observed in 2020, coinciding with warming and humidification trends (Wang et al. 2025; Ban et al. 2022).

However, habitat expansion at the periphery is accompanied by challenges in core areas, where warming may be pushing conditions beyond the species' physiological tolerances or altering habitat quality. Our results show that the expansion of suitable habitats is accompanied by habitat loss in some areas (Figure 4). This dual phenomenon‐habitat gain at range edges and loss in central areas—has also been observed in high‐altitude specialists such as the Himalayan Monal, where core habitat contractions occur despite overall distribution increases (Lochan et al. 2025). These patterns reflect the complexity of climate‐induced habitat dynamics and underscore the need to manage both expanding and contracting habitats.

### 4.2 Changing Environmental Drivers

We compared the key environmental drivers of black‐necked crane distribution in 2006 and 2020 and found a marked transformation in habitat determinants, and found that the determinants of habitat suitability have undergone a marked transformation. This is further evidenced by the shift in variable importance scores from 70.7% LULC in 2006 to 56.9% in 2020, with climate variables such as G‐bio8 and G‐bio18 gaining prominence.

In 2006, habitat suitability was primarily driven by LULC, reflecting strong human influence. Fallow farmlands provided key food resources during winter, and wetland conversion to cropland reinforced the species' dependence on agricultural landscapes (Wu et al. 2020). Limited protected area coverage and expanding land use likely intensified this pattern (Wang, Wang, et al. 2021). By 2020, the importance of climatic factors (such as precipitation and temperature) had gradually increased, whereas the importance of LULC significantly decreased (Table 2). climatic factors—particularly precipitation and temperature—became more influential. This coincides with warming and humidification trends across the QTP, which have expanded wetlands and boosted vegetation productivity (He et al. 2022; Liu et al. 2022). Seasonal rainfall, especially in summer, shapes water availability and food resources, strengthening climate variables' role in habitat suitability (Wu et al. 2020).

The reduced importance of LULC may reflect the positive effects of conservation policies, such as wetland restoration, farmland management, and ecological compensation, which have alleviated direct human pressures. In some regions, land‐use impacts may have been moderated by programs like Payments for Wetland Ecosystem Services (PWES) in Dashanbao (Peng 2020). These interventions may reduce LULC's influence in SDMs, allowing broader‐scale climatic drivers to dominate. However, this remains a hypothesis that requires more detailed field‐level and social‐ecological studies. For black‐necked cranes, precipitation during the warmest quarter supports vegetation growth, whereas precipitation during the coldest quarter ensures roosting wetland availability (Wu et al. 2020). These changes may drive ecological niche drift, reflecting broader responses to rapid warming and shifting precipitation regimes. Comparable shifts have been documented in other wetland‐dependent species. For instance, Barredo et al. (2016) found that rainfall in comparison with temperature, increasingly determined the distribution of wetland birds in Mediterranean climates. (Barredo et al. 2016). Similarly, migratory shorebirds have shown growing reliance on precipitation patterns over temperature extremes for habitat selection (Steen et al. 2018). This transition underscores the evolving ecological drivers of species distribution in the face of climate change.

Although our findings are broadly consistent with previous research, they are limited by the lack of detailed field investigations, small data size, and the limited number of study years. Big data models are powerful for large‐scale analysis but may overlook fine‐scale ecological processes, such as food availability, which are critical for habitat selection. Multi‐scale approaches are essential for a comprehensive understanding of black‐necked crane habitat use. Future studies should integrate ecological, behavioral, and socio‐environmental data—such as migration pathways, foraging behavior, and human land‐use patterns—into SDMs to refine conservation strategies. Addressing both climatic and anthropogenic threats will strengthen efforts to protect this flagship species and the fragile ecosystems it represents.

### 4.3 Conservation Implications

The findings underscore the urgency of integrating climate adaptation strategies into conservation planning for black‐necked cranes. These results align with broader conservation literature emphasizing the need for adaptive strategies in rapidly changing ecosystems (Bhakti et al. 2018; Turner et al. 2007).

Protecting core habitats, such as the Yarlung Zangbo River basin and Qinghai Lake, is essential for sustaining existing populations. Simultaneously, managing human impacts in expanding areas, including agricultural landscapes, is critical. Implementing landscape corridors can maintain ecological connectivity and reduce the risks of fragmentation (Bhakti et al. 2018). Protecting wetlands and ensuring natural water flow in critical habitats are vital to buffer the impacts of climate change. For example, proactive water resource management along the Yarlung Zangbo River basin and Qinghai Lake could sustain vital wintering grounds under variable climate conditions.

## 5 Conclusions

This study examined the changes in the suitable habitats of the black‐necked cranes over the past 15 years and identified shifts in the dominant environmental drivers. Our findings reveal a transition from LULC‐driven habitat suitability to increasing importance of climatic factors, reflecting broader ecological changes on the Qinghai‐Tibet Plateau. Although warming temperatures have enabled habitat expansion, particularly toward higher altitudes and latitudes, concurrent losses and fragmentation of core habitats highlight emerging conservation challenges. To ensure the long‐term survival of black‐necked cranes, conservation efforts should prioritize safeguarding core habitats, managing newly suitable areas, and maintaining ecological connectivity through landscape corridors. Adaptive water resource management is essential to mitigate the impacts of climate variability. Our findings highlight the need for dynamic conservation strategies that anticipate climate‐driven habitat shifts and support ecological resilience across the Tibetan Plateau.

## Author Contributions

**Le Yang:** conceptualization (equal), data curation (equal), formal analysis (equal), methodology (equal), visualization (equal), writing – original draft (equal), writing – review and editing (equal). **Ting Wu:** data curation (equal), methodology (equal), visualization (equal), writing – original draft (equal), writing – review and editing (equal). **Waner Liang:** data curation (equal), investigation (equal), writing – original draft (equal), writing – review and editing (equal). **Qin Zhu:** data curation (supporting), methodology (supporting), visualization (supporting), writing – original draft (supporting). **Lei Xu:** investigation (equal), resources (equal). **Jia Guo:** investigation (equal), resources (equal). **Cai Lu:** investigation (equal). **Qing Zeng:** data curation (equal). **Mary Anne Bishop:** investigation (equal). **Li Wen:** conceptualization (equal), data curation (equal), formal analysis (equal), methodology (equal), software (equal), visualization (equal), writing – original draft (equal), writing – review and editing (equal). **Yifei Jia:** conceptualization (lead), data curation (equal), funding acquisition (equal), investigation (equal), methodology (equal), project administration (lead), resources (equal), supervision (equal), visualization (equal), writing – original draft (equal), writing – review and editing (equal). **Guangchun Lei:** writing – review and editing (equal).

## Funding

This work was supported by Yifei Jia, Grant Number: 2019QZKK0304, Grant Number: XZ202201ZY0005G, Grant Number: XZ202301YD0007C.

## Conflicts of Interest

The authors declare no conflicts of interest.

## Supporting information


**Table S1:** All the environmental variables.
**Table S2:** Summary of model characteristics and evaluation measures. For each model, AUC (area under the curve of the receiver operating characteristic), TSS (mean true skill statistics), and Boyce Index for the test dataset are reported.
**Figure S1:** Response curves of the black‐necked crane to environmental factors in 2006.
**Figure S2:** Response curves of the black‐necked crane to environmental factors in 2020.

## Data Availability

The data that support the findings of the study can be obtained at https://doi.org/10.5061/dryad.sj3tx96h5.
