# Supplementary material for: Rising Influence of Climate on the Distribution of Black‐Necked Cranes (Grus nigricollis) on Tibetan Plateau
Source: Ecol Evol. 2025 Dec 28;15(12):e72756. doi: 10.1002/ece3.72756 (PMC12744892; doi:10.1002/ece3.72756)
Supplement: Supplementary file 1 — Table S1: All the environmental variables. Table S2: Summary of model characteristics and evaluation measures. For each model, AUC (area under the curve of the receiver operating characteristic), TSS (mean true skill statistics), and Boyce Index for the test dataset are reported. Figure S1: Response curves of the black‐necked crane to environmental factors in 2006. Figure S2: Response curves of the black‐necked crane to environmental factors in 2020. [file ECE3-15-e72756-s001.docx]

**Table S1**. All the Environmental Variables

| No. | Environmental Variables | Description |
| --- | --- | --- |
| 1 | G-bio1 | Annual Mean Temperature |
| 2 | G-bio2 | Mean Diurnal Range |
| 3 | G-bio3 | Isothermality |
| 4 | G-bio4 | Temperature Seasonality |
| 5 | G-bio5 | Max Temperature of Warmest Month |
| 6 | G-bio6 | Min Temperature of Coldest Month |
| 7 | G-bio7 | Temperature Annual Range |
| 8 | G-bio8 | Mean Temperature of Wettest Quarter |
| 9 | G-bio9 | Mean Temperature of Driest Quarter |
| 10 | G-bio10 | Mean Temperature of Warmest Quarter |
| 11 | G-bio11 | Mean Temperature of Coldest Quarter |
| 12 | G-bio12 | Annual Precipitation |
| 13 | G-bio13 | Precipitation of Wettest Month |
| 14 | G-bio14 | Precipitation of Driest Month |
| 15 | G-bio15 | Precipitation Seasonality |
| 16 | G-bio16 | Precipitation of Wettest Quarter |
| 17 | G-bio17 | Precipitation of Driest Quarter |
| 18 | G-bio18 | Precipitation of Warmest Quarter |
| 19 | G-bio19 | Precipitation of Coldest Quarter |
| 20 | SMI | Soil Moisture Index |
| 21 | LULC | Land Use and Land Cover |

**Table S2.** Summary of model characteristics and evaluation measures. For each model, AUC (area under the curve of the receiver operating characteristic), TSS (mean true skill statistics), and Boyce Index for the test dataset are reported.

|  | **Period** | **AUC** | **TSS** | **Boyce Index** |
| --- | --- | --- | --- | --- |
| Black-necked Crane | 2006/2007 | 0.945 | 0.809 | 0.547 |
|  | 2020/2021 | 0.972 | 0.881 | 0.500 |

**Table S3. The gain and loss of Wintering Black-neck Crane in QTP between 2006 and 2020**

|  | loss | pre | abs | gain |
| --- | --- | --- | --- | --- |
| Area (km²) | 3370 | 6850 | 279840 | 17460 |


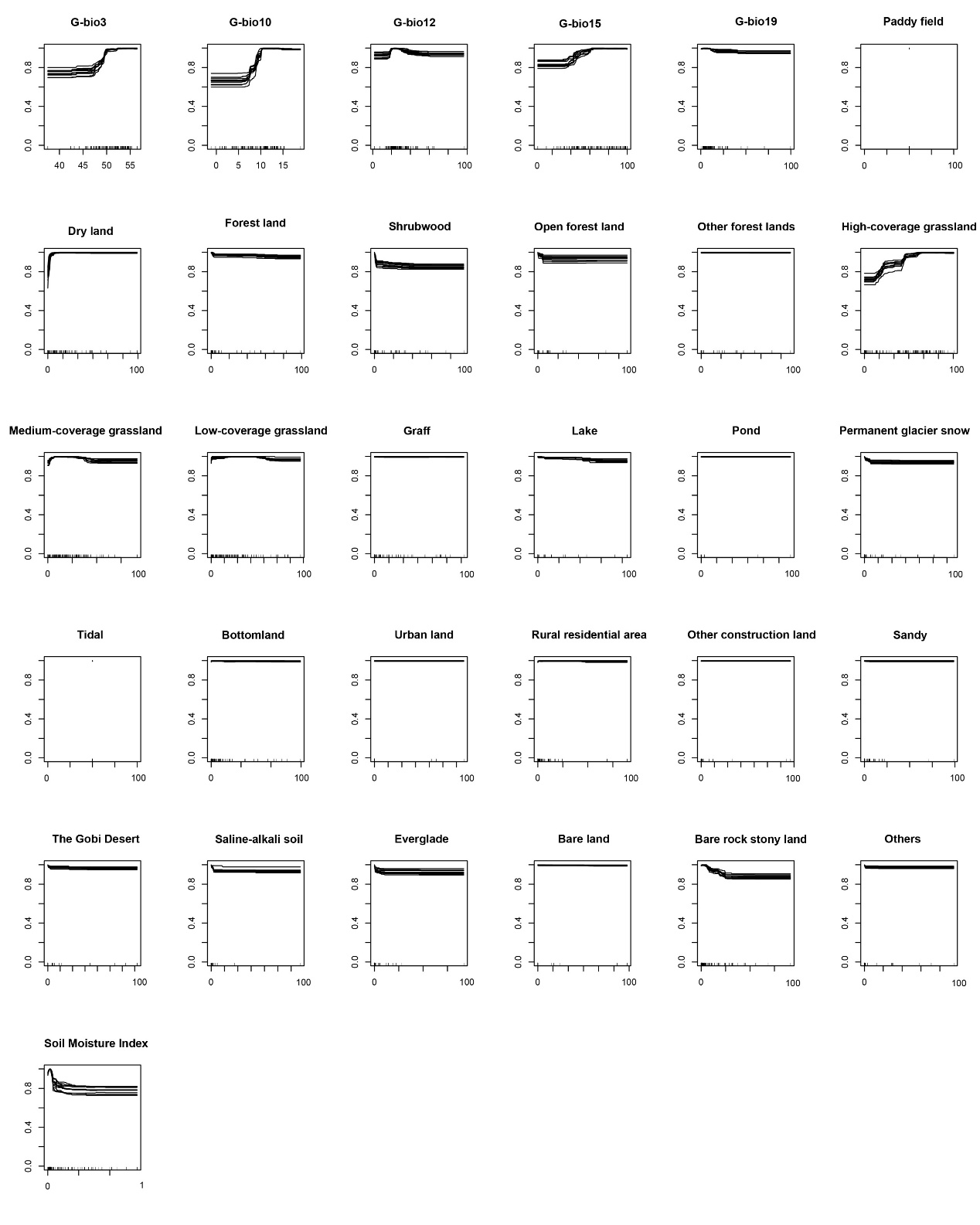


**Fig. S1. Response curves of the Black-necked Crane to environmental factors in 2006.**


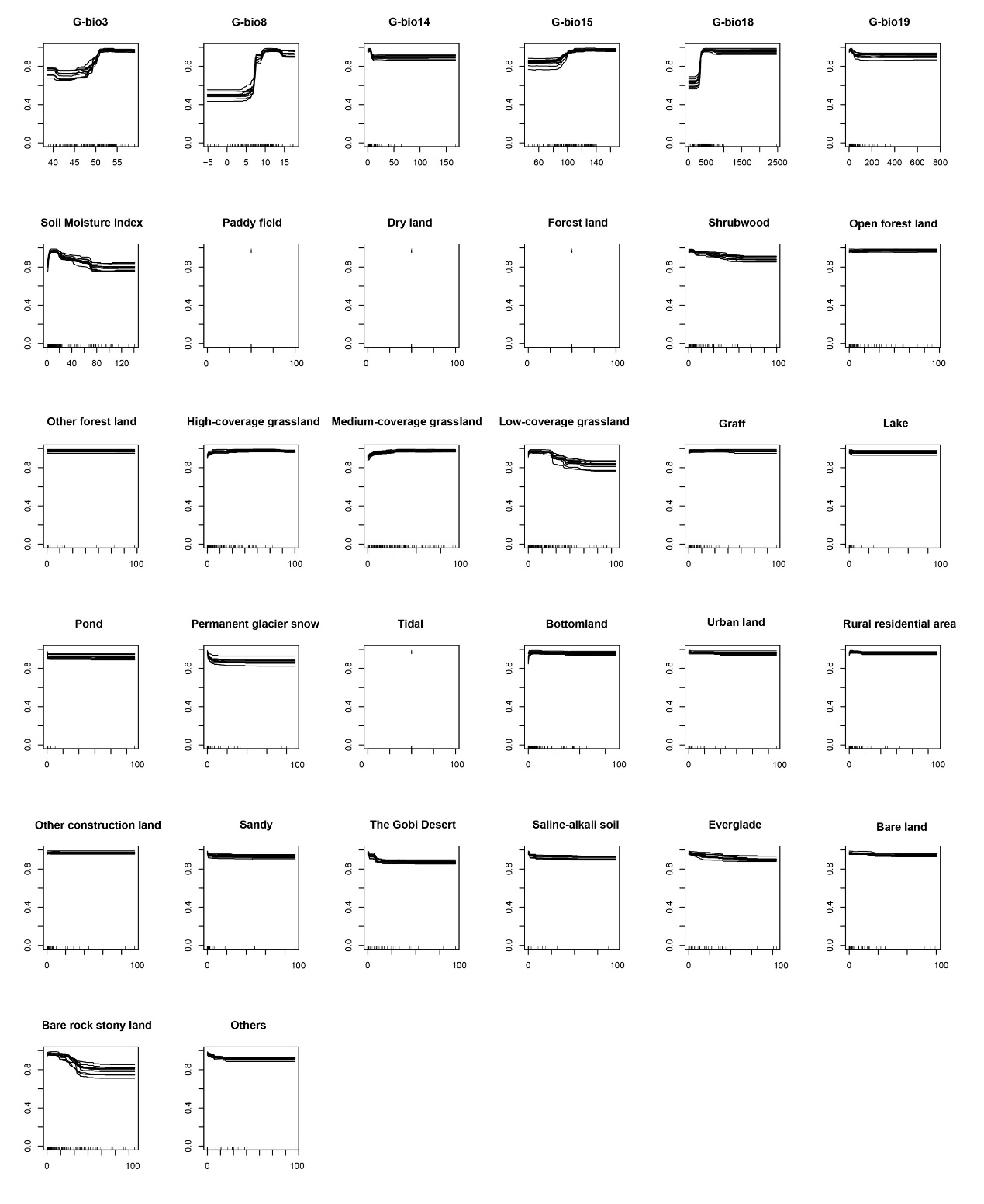
**Fig. S2. Response curves of the Black-necked Crane to environmental factors in 2020.**
